# Effect of Supplementation of Fermented Yeast Culture on Hormones and Their Receptors on Exposure to Higher Temperature and on Production Performance after Exposure in Nicobari Chickens

**DOI:** 10.1155/2021/5539780

**Published:** 2021-08-03

**Authors:** A. L. Nidamanuri, Lawrence Leslie Leo Prince, S. P. Yadav, T. K. Bhattacharya, S. R. R. Konadaka, S. K. Bhanja

**Affiliations:** Directorate of Poultry Research, Rajendranagar, Hyderabad, Telangana State, India

## Abstract

Heat stress (HS) affects the production performance in chickens and causes economic loss to the producers. Most of the studies have been conducted on and for the welfare of broilers. We still lack information on the physiological parameters being affected during chronic heat stress in layers. To fill this gap, the present study evaluated the effect of heat stress (induced in the chamber) during the prelaying period (21–23 weeks) on plasma levels of the hormones leptin and ghrelin and GH and expression of the respective receptors and heat stress markers. Three groups were considered, one at room temperature (CR) and the other two groups (SH and CH) subjected to heat stress at 39°C for four hours for three weeks (21–23 weeks of age). The SH group (SH) feed was supplemented with fermented yeast culture (FYC, 700 mg/kg), whereas the CH group was devoid of it. After that, all the groups were shifted to shed under natural ambient conditions till 31 weeks of age. Studies were restricted to production performance only. Feed offered without yeast culture (CH group) had a smaller concentration of plasma hormones (*P* < 0.01) and increased expression fold of the hormone receptors (*P* < 0.01). Further, the group also presented higher liver AMP kinase enzyme, plasma MDA (malondialdehyde), and cholesterol concentrations. These changes likely explained the decrease in feed intake and the CH group's body weight and further reduced the production performance during the laying period. Supplementation with FYC to birds had an opposite effect on the above-mentioned parameters, reducing HS effects. In summary, supplementation with FYC (700 mg/kg) maintained physiological parameters as in the CR group under HS conditions and negated adverse effects on parameters both before and during laying periods.

## 1. Introduction

The consequences of heat stress (HS) on chickens attract many researchers' attention [1, 2]. Nowadays, we know the impact on the physiological parameters, notably, increased body core temperature, reduced feed intake, alteration of endocrine, and reproductive functions. Heat stress (HS) causes enormous economic losses in the poultry industry [3, 4]. If the temperature rises above the critical threshold, the birds experience stress, thus altering their physiological functions. Besides, constant high temperature becomes more deleterious to birds than cyclic or alternating temperatures [5]. It leads to decreased feed intake and egg production and higher mortality [6, 7].

In the last two to three years, a number of reports with respect to the amelioration of heat stress are available. Reports are based on amelioration of heat stress by supplementation of herbal products, prebiotics, postbiotics, probiotics, minerals, enzymes, and vitamins [8] to chickens [8–16]. Otherwise, also there are a number of feed additives routinely used in poultry feed, such as antibiotics, probiotics, oligosaccharides, enzymes, and organic acids [17, 18], for promoting growth through their potential effect in increasing feed intake [19, 20]. Most of the work is concentrated on the welfare of broilers.

A study on broilers reported that chronic heat exposure significantly increased cloacal temperatures and respiration rates when compared to the control group. Even in layer reports that are available with respect to amelioration of heat stress, they have concentrated on the effects of supplement only during the laying period. The present work describes the effect of applied heat stress (HS) during prelaying on the physiological parameters, especially the effect on mentioned hormones and their receptors, which has not been reported, and their posteffect in terms of decreased production performance has also been not reported. The present study reports that a supplement like FYC which has not been used for alleviation of heat stress is able to increase the production performance by reversing the effects of HS through modulation of hormones, metabolites, enzymes, and gene expression.

Additionally, it is reported that heat stress elevated ghrelin concentrations in serum and the intestine [21]. In mammals, ghrelin is usually described as an orexigenic hormone whose concentration increases during calorie restriction [22]. In chickens, ghrelin plays a distinct role. Exposure to heat decreased the plasma ghrelin level [23]. The short- and long-term environmental heat affect endocrine glands and, in turn, hormones, which further causes a decline in plasma levels after prolonged exposure. The reports regarding the effects of heat stress on hormones in broilers are many, but studies reflecting the impact on the mentioned parameters in layers are lacking. Another essential parameter affected by heat stress is the AMP kinase (AMPK) enzyme. The AMPK is one of the primary cellular energy sensors [24] and an intracellular low-energy warning system [25].

Heat stress induces an increase in intestinal permeability that is associated with massive generalized sloughing of epithelial layer from the villus tips and lysis of intestinal epithelial cells, indicating that the increase in intestinal permeability is due to the extensive damage of the epithelial surface [26]. Yeast cell walls are beneficial as natural feed additives and are widely used in poultry diets [27–29]. Yeast culture (YC) is a natural yeast fermentation product that includes yeast cells, vitamins, peptides, amino acids, and proteins [30]. Some other feed additives are also used to alleviate heat stress, such as trace elements, vitamins, and probiotics [31].

Liver plays an important role in digestion and metabolism, regulating the production, storage, and release of lipids, carbohydrates, and proteins [32]. In chickens, leptin is expressed mainly in the liver, where its receptor gene expression has also been reported, and in adipose tissue [33].

Magnum is one of the regions in the chicken oviduct which produces egg white. Though 88% of the albumen is water, this component of the egg contributes more than 60% to the total egg weight which is an essential quality of an egg [34]. Hrabia et al. [35] have demonstrated differential mRNA expression and protein localization of GH receptors in the infundibulum, magnum, isthmus, and shell gland of laying hens.

Nicobari is one of the indigenous breeds of chickens [36]. Nicobari fowl is an endangered breed of the Andaman and Nicobar Islands and produces the highest number of eggs among India's indigenous chicken breeds [37]. Hence, it becomes crucial to study physiological parameters' modulation when these birds are subjected to heat stress.

The current study hypothesizes that heat stress during the prelaying period affects production performance during the laying period. Continuous exposure to higher ambient temperature under controlled conditions is more effective compared to normal varying temperatures and may modulate the level of plasma hormones, metabolites, enzymes, and tissue gene expression. A supplement like FYC may be beneficial by removing the adverse effects of heat stress on the mentioned parameters, during the prelaying period (heat stress under controlled conditions) by modulating physiological parameters, and further on ovarian functions during the natural environmental conditions.

## 2. Materials and Methods

### 2.1. Animals and Experimental Setup

Nicobari chickens were maintained at an experimental poultry farm, ICAR-Directorate of Poultry Research, Rajendranagar, Hyderabad, India. The Institutional Animal Ethics committee approved all experimental procedures conducted in this study. At the beginning of the experiment, birds of 18 weeks of age were randomly selected from the farm and maintained in individual cages with four to five chickens in a group in a cage. The cages were arranged in 3 tiers with two cages per tier and were placed on wheels. Before the experiment began, the birds were placed in the cages for one week to acclimatize to the new surroundings. From week 19 till the beginning of the 21st week, birds were gradually acclimatized to higher temperatures by raising the temperature from 37 to 39^o^C. Three groups of birds were maintained. The number of birds in each group was thirty. Each group had five replicates with six birds in each group. The first group was the control group (CR), which was maintained at room temperature, the temperature varying between 22 and 26^o^C during the daytime. The second (CH) and third (SH) groups were exposed to a controlled temperature chamber continuously at 39^o^C for four hours (11 am to 3 pm) during three weeks starting from 21 weeks of age till the birds attained 23 weeks of age. CH group was offered feed devoid of supplement FYC, and SH was provided with FYC (Unigrow, Nurture Organics, New Delhi) at 700 mg/kg feed according to the manufacturer's instructions. The dose was standardized based on a pilot study conducted at our farm. The amount of feed in the form of mash (based on maize and soya, Table 1) [38] provided was 90 g/bird/day, and water was provided ad libitum. After exposure to 39^o^C temperature, they were exposed to cyclic ambient room temperature. The humidity in the chamber was adjusted to 50% during the experiment. A regime of 16 h light was provided and all the chickens were kept under uniform light management conditions.

Initially, the chamber's temperature was increased to 32°C; birds of CH and SH groups, placed in the cages, were shifted to the chamber where the temperature was set to 32°C. Further, the temperature was raised to 39°C at a rate of 2°C for every half an hour. The birds were then maintained at 39°C for four hours. Similarly, at the end of the exposure, the temperature was brought down slowly to 32°C, and then the birds were shifted to a room at ambient temperature. Care was taken to avoid spillage of feed and water. After moving them to room temperature, the water was replaced with normal drinking water. Rectal temperature was recorded with the help of a clinical thermometer. The thermometer was introduced into the cloaca 1.5 cm deep inside and allowed to stabilize before recording temperature. In each experiment, temperature values were recorded from five birds at random of each experimental unit for twenty-one days at 9 am and 4 pm, respectively. Exposure time used to get over by 4 pm.

### 2.2. Estimation of Body Weight, Feed Intake, and Egg Production Parameters

Body weight was recorded weekly for ten birds taken at random from 21 to 23 weeks of age during the course of exposure and further average value was estimated for fortnight interval starting from 24 weeks till the birds attained 29 weeks of age after exposure to a higher temperature. After three weeks of exposure, the birds were shifted to individual cages, and then they were exposed to ambient room temperature during summer in the shed.

Feed refusal was recorded every week to calculate feed intake every day, and average feed intake/bird/day for a week was estimated. From 25 to 29 weeks of age, average feed intake (FI) was estimated for 14-day intervals and expressed as feed intake/bird/day. The ambient temperature remained at the peak from 11.00 am to 3.00 pm, and peak temperature varied from 32 to 36°C (March-April) in the shed. The study on egg production continued till 30 weeks of age, where the effect of controlled heat stress during the prelaying period was observed during the laying period. Further, the effect of supplementation was also evaluated. Supplementation continued after exposure also, during the laying period to the SH group till 30 weeks of age. The number of eggs laid and the eggs' weight were recorded every day for the three groups.

### 2.3. Collection of Blood Samples, Processing, and Estimation of Plasma Parameters

At 10 and 20 days from the beginning of the experiment, at the end of exposure to 39°C, 1.5–2 ml of blood was drawn from the wing vein of six birds from each group into EDTA rinsed tubes. The blood was immediately centrifuged at 3000 rpm for 12 min. The plasma was separated and stored at −20°C to further analyze leptin (E12L0038) and ghrelin (E12G0013) hormones and GH (E12G0165), using commercial EIA kits (BlueGene Biotech, chicken Elisa kits, Shanghai, China); intra- and interassay variation was <7%. Plasma cholesterol (E03C0745), MDA (E03M0023), and liver AMP kinase (E12P0187) enzyme concentration were analyzed using commercial kits procured from BlueGene Biotech Co., Shanghai, China. The mentioned parameters were estimated according to the manufacturer's protocol.

### 2.4. Collection of Tissue Samples and Processing for Quantification of Different Parameters

Liver, magnum, and jejunum tissue samples were collected from randomly selected birds from each group and processed as described by Anand Laxmi et al. [39] after sacrificing five birds from each group at 10 and 20 d of the experiment. Chicken liver and magnum samples were processed for studies on the expression of leptin receptor (LEPR), ghrelin receptor (GSHR), and growth hormone receptor (GHR), whereas histological studies were conducted on jejunum samples to observe the severity of necrosis.

Magnum is a region of the oviduct extending between infundibulum and isthmus. Liver tissue was also processed for estimation of the concentration of enzyme AMP kinase, which was analyzed using commercial kits procured from BlueGene Biotech Co., Shanghai, China. Enzyme was estimated according to the manufacturer's protocol.

Jejunum samples for the histological study were processed as given by Quinteiro-Filho et al. [40]. Jejunum region extends from the duodenal loop to Meckel's diverticulum. Briefly, for histopathological studies on jejunum, the jejunum portion of the digestive tract was excised and cleaned from the sacrificed birds of each group. The contents of the jejunum were gently removed. The tissue was fixed in 10% formaldehyde. Samples were kept in Bouin's solution and dehydrated in a graded series of alcohol. Finally, after embedding each specimen in paraffin wax, sections were fixed and stained with hematoxylin-eosin. Further, the slides were observed under a light microscope at 400x magnification to observe the villus' morphology and necrosis. The severity of the necrosis was graded as medium (++), mild (+), and normal (±).

### 2.5. Isolation of RNA and Quantitative Real-Time PCR

Total RNA was isolated from liver and magnum tissue (100 mg approx.) using Trizol. cDNA was synthesized using 1 ug of RNA with the help of a First-Strand cDNA kit (Thermo Fisher Scientific Co.). The primers were got synthesized by Chromous Co., Bangalore, India (Table 2). Before proceeding on to real-time PCR, the primers were tested for the formation of respective gene products by the standardized PCR protocol [39] as conducted previously.

Further qPCR or gene expression studies for hormone receptors in the liver and magnum were also conducted. The messenger RNA (mRNA) expression of target genes was evaluated by real-time PCR. Real-time quantitative PCR was conducted using the SYBR® Green dye method and the SYBR Premix (InCell Technologies, Hyderabad) on a 7500 real-time PCR instrument (ABI, USA). The actin gene was utilized as a housekeeping gene to normalize the expression data of the target genes. The PCR program was operated as follows: 95°C for 60 s, 40 cycles of 95°C for 30 s, and 58°C for the 30°s. Each reaction for a gene was repeated three times. The relative gene expression was calculated using the 2−ΔΔ*C*T method [41].

Studies on hormone levels and gene expression studies were restricted to three weeks (the age of the birds was 21–23 weeks). Comparison of gene expression studies for hormone receptors was restricted to the CH and SH groups only. Further, after exposure trials, they were shifted to individual cages in the farm, where they were exposed to ambient cyclic room temperature during summer, which coincided with the birds' laying period. The effect of high ambient temperature (under controlled conditions) during the prelaying period was observed on body weight and egg production from 24 weeks onwards, which was the postexposure period during the summer season in the poultry shed.

### 2.6. Statistical Analysis

The effects of three treatments heat stress with and without supplement and also negative control (without stress and supplement) on hormones, receptors, and metabolites were done using one-way ANOVA. Birds were randomly assigned and selected from the different groups for the experiment and sampling purposes. Data were analyzed by analysis of variance (ANOVA) using the general linear model procedure (GLM) of SPSS 12 for Windows. LSD was used for pairwise comparison of the least square means *P* < 0.05 [42].

## 3. Results

### 3.1. Cloacal Temperatures and Plasma Hormones

The average cloacal temperatures recorded from 21 to 23 weeks of age at 9 am at R.T. in the shed were 41.2, 41.3, and 41.2°C, whereas at 5 pm at the end of the exposure, the temperatures were 41.4, 42.3, and 42.4°C for the CR, CH, and SH groups, respectively. The analysis of plasma leptin hormone revealed that plasma concentration in the CH group was significantly less (*P* < 0.01) when compared to CR (−69.8% on day 10 and −50.5% on day 20) and SH (−72.8% on day 10 and −55.7% on day 20) (Table 3). Besides, the concentration of ghrelin hormone was significantly (*P* < 0.01, Table 2) less at both 10- and 20-day intervals when compared to the values in the CR (−50.2% and −26.2%) and SH group (−50.9% and −12.45%). The statistical difference was observed on day 20 only, being greater in the CR group (Table 2). The plasma GH hormone analysis revealed that both the CH and SH groups showed higher concentrations than the CR group at the 10-day interval. However, at the 20-day intervals, it was observed that the values in CH dropped to 2.83 ± 0.11 that represented a significant reduction (*P* < 0.01) when compared to the values in CR (−23.7%) and SH (−23.5%) (Table 3).

### 3.2. Plasma MDA and Cholesterol

The supplementation of FYC reduced plasma MDA levels (*P* < 0.01; Table 3). The SH group presented −40.8% plasma MDA concentration in relation to the CH group and −27.2% with regard to the CR group on day 10. Similar results were also found on day 20, where the SH group presented 30.7% less plasma MDA than the CH group and 6.0% less than the CR group. Supplementation of FYC also contributed to a change in the levels of plasma cholesterol (Table 3). On day 10, the SH group showed 25.8% and 25.2% more cholesterol than the CH and CR groups, respectively. However, mean ± SE values for day 20 revealed that the cholesterol concentrations in SH did not differ statistically from the CR group and reduced 13.1% concerning the CH group.

### 3.3. Liver AMP Kinase Enzyme

Further upon analysis of liver AMP kinase enzyme, levels did not show any statistical difference between the three treatments on day 10. However, at 20 days, it was observed that the FYC treatment reduced AMP kinase levels by 22.4% compared to the CH and 13.6% compared to the CR treatment groups (*P* < 0.05; Table 3).

### 3.4. Production Performance

The supplementation with FYC affected the body weight (BW) of Nicobari chickens, but statistical differences became detectable after 22 weeks of age (Table 4). The difference in BW between the CH and CR groups was not significant at any age. Compared to CH, the SH treatment represents a gain in BW that ranged between 3.9 and 6.1%, whereas, compared to CR treatment, the BW gain ranged between 4.7 and 5.8% from 22–29-week-old chickens. Heat stress significantly reduced the feed intake (*P* < 0.01) in the CH group when compared to control treatment (CR). However, when Nicobari chickens (SH) received the supplementation, the feed intake was similar when compared to control treatment (CR) at 21 and 27 weeks but was significantly higher at other time intervals (22‐25) (Table 5). Besides, supplementation with FYC increased the feed intake under heat stress.

Further, the study evaluated the effect of high ambient temperature on egg production (postexposure) from 23 to 30 weeks. Egg production started early in the SH group (23rd week), where an average of 6.6% of Nicobari chickens began to lay eggs, whereas the other two groups started later (CR = 24th week; CH = 25th week; Table 6). Age at sexual maturity also showed the same trend (161 < 168 < 175 days). The percentage of egg production at 30 weeks for different groups was 70.32 > 59.63>57.23 (SH > CR > CH). The rate of egg production was less in the CH group when compared to the other two groups.

### 3.5. Histological Studies

**Figure 1:**
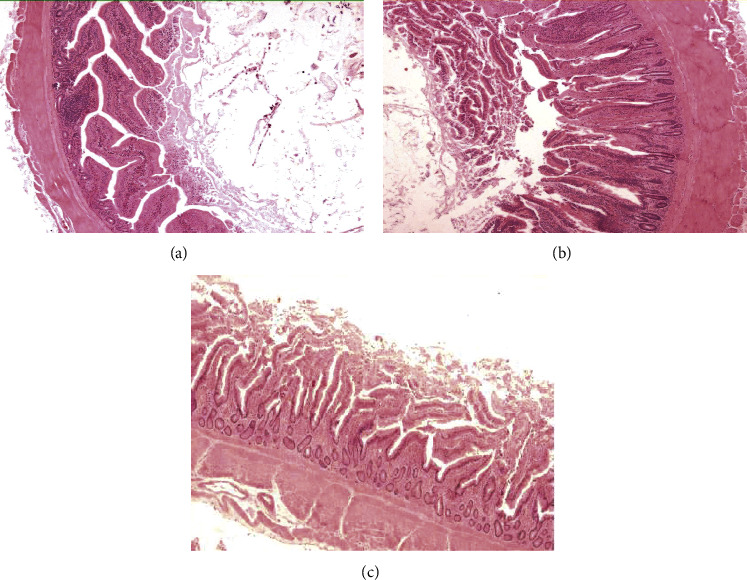
Histomorphology of the jejunum exhibiting. (a) Normal villi, (b) mild (+), and (c) medium (++) necrosis. Magnification: 400x.

In the present experiment, mild (+) to medium (++) necrotic condition of villi was observed in all groups of birds; percentage exhibiting mild necrosis was observed to be 80 and 85% in the CR and SH groups, respectively, when compared to the CH group (30%). In contrast, the percentage of villi with medium necrotic effect (70%) was observed more often in the CH group at both time intervals (Figure 1). The percentage of normal villi and villi with medium necrosis effect was 10 and 5% in CR and SH groups of chickens, respectively.

### 3.6. Gene Expression Studies

**Figure 2:**
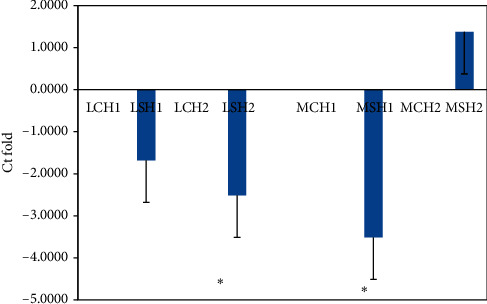
Comparison of relative fold change in the gene expression of leptin receptor (LEPR) in liver (L) and magnum (M) tissues of Nicobari chicken after 10 d (CH1 and SH1) and 20 d (CH2 and SH2) of the experiment. ^*∗*^*P* < 0.05. C: control; S: supplemented with FYC at 700 mg/kg *N* = 4. From 21 to 23 weeks of age, the CH and SH group birds were exposed to 39°C for 4 h under controlled conditions.

**Figure 3:**
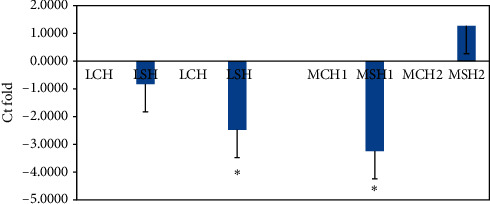
Comparison of relative fold change in the gene expression of ghrelin receptor (GSHR) in liver (L) and magnum (M) tissues of Nicobari chicken after 10 d (CH1 and SH1) and 20 d (CH2 and SH2) of the experiment. C: control; S: supplemented with FYC at 700 mg/kg. ^*∗*^*P* < 0.05*N* = 4. From 21 to 23 weeks of age, the CH and SH group birds were exposed to 39°C for 4 h under controlled conditions.

**Figure 4:**
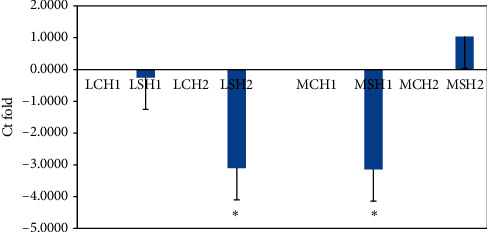
Comparison of relative fold change in the gene expression of ghrelin receptor (GHR) in liver (L) and magnum (M) tissues of Nicobari chicken after 10 d (CH1 and SH1) and 20 d (CH2 and SH2) of the experiment. C: control; S: supplemented with FYC at 700 mg/kg. ^*∗*^*P* < 0.05*N* = 4. From 21 to 23 weeks of age, the CH and SH group birds were exposed to 39°C for 4 h under controlled conditions.

Expression of all hormone receptors was downregulated in the liver and magnum tissue of the supplemented group when compared to the CH group on day 10 (Figures 2–4 ), but on day 20, it was upregulated in the magnum tissue (Figures 2–4). On day 10, the difference in the fold change in the expression of hormone receptors of liver tissue between the CH and SH groups was not significant; however, it was significant (*P* < 0.05) only on day 20; it was significantly downregulated in the SH group.

## 4. Discussion

In the present experiment, the average cloacal temperatures of Nicobari pullets subjected to heat stress increased by 0.92^o^C (CH) and 1.02^o^C (SH) over that of the CR group of birds. Chickens present an internal temperature of 41^o^C, but an increase of 4^o^C or more above is considered fatal [43]. Basilio et al. [44] observed that, with an ambient temperature between 38 and 40°C and relative humidity between 50 and 55%, a chicken's rectal temperature could be elevated to 45–48°C leading to death by heat stress and a decrease in production efficiency. Hence, the present study was conducted in Nicobari layers. Since India is a tropical country, and Nicobari is a native bird, continuous exposure to 39°C for 4 h might not have caused the rise in temperature, as reported in the earlier references. The mortality rate was 1% in the CH group, whereas in the other two groups, it was 0.5%. Even then, we observed the effects of heat stress on the different parameters, such as a decrease in feed intake, body weight, and production of eggs of the CH group. Similar reports are available [10, 11].

Exposure to high temperature (39°C for 4 h) decreased the mean level of hormones in the Nicobari birds when compared between the CH and CR groups. However, supplementation with 700 mg/kg of FYC could restore or increase the level of respective hormones in the SH group of birds. It was hypothesized that the level of hormones in the CR group was optimal for functions. The results obtained on days 10 and 20 were different. Mean levels of plasma leptin and ghrelin were significantly less in the CH group at both days 10 and 20, whereas the level of plasma GH was less only on day 20 when compared to respective hormone levels of SH and CR groups. Even though the plasma leptin level increased on day 20, it was still less when compared to the other two groups. Supplementation with FYC maintained the levels of leptin as observed in the CR group. This finding indicates that supplementation of FYC reduced the influence of heat stress on the levels of plasma leptin.

With respect to ghrelin hormone, supplementation of FYC could maintain a similar level only on day 10, but not on day 20, when compared to the CR group. Exposure to heat induces a decrease in plasma ghrelin levels [23]. In the CH group, even though the hormone level increased on day 20 compared to the level on day 10, it was still less compared to levels of plasma ghrelin in the other two groups. Ghrelin is known to stimulate feed intake in mammalian species while considering an anorexigenic hormone in chickens [45, 46]. In the present study, the relationship between the plasma levels of ghrelin and leptin on feed intake observed was not inversely related and the effect appeared to be independent. Instead, a decrease in FI became significant at a later stage during the postexposure period in the CH group, which is considered a delay in effect on FI. Ghrelin modulates the response to food cues via a neural network involved in regulating feeding and the appetitive response to food cues. Exposure to higher temperature increased plasma GH level significantly in both CH and SH groups when compared to CR, indicating that the effect of FYC on GH at this time interval was not significant; but at day 20, the plasma GH level in the supplemented group was observed to be similar to the hormone levels observed in room temperature maintained birds. The GH level observed in room temperature birds was not significantly different from day 10. Hence we can say that GH hormone level even though decreased in SH and CH groups, the level was comparable to the CR group. Hence supplementation of FYC was beneficial. However GH decreased significantly in CH group also, which was significant when compared with the other two groups. Chronic heat stress downregulated the mRNA expression of GH in liver [47]. The decrease in the level of all the hormones at 10 or 20 d of the CH group might have contributed to decreased production performance.

Supplementation of FYC caused the downregulation of expression of the receptors, LEPR, GSHR, and GHR for all the hormones in both liver and magnum tissues indicating reduced cellular activity. When the number of receptors decreases in response to rising hormone levels, called downregulation, cellular activity is reduced ([48, 49]. In the present experiment, the increase in hormone levels in the SH group might have downregulated the hormone receptors. The reduction in cellular activity might have also caused less energy requirement, bringing down the birds' metabolic activity. In the CH group, the opposite effect was observed on expression of receptors. It indicated the necessity of greater energy in these birds under heat stress [19]. The up- and downregulation of the receptors may be for maintaining homeostasis of the system [50]. In humans, it was observed that when there is an inflammatory condition of the liver, it may result in increased energy expenditure and reduced nutrition intake [51, 52]. Similar results were obtained in the present study. Ghrelin receptors have been localized in the liver of chickens [53]. The expression pattern of ghrelin and its receptor mRNAs was observed to change in liver depending on feeding states in poultry [54]. Since GH receptors have been localized in all regions of the oviduct and treatment of prelaying hens with cGH increased ovalbumin expression in magnum [35, 55], it can be suggested that an increase in plasma GH in FYC supplemented group during HS might have increased the function of magnum and other regions leading to increase in egg production. The same authors have reported that GH induces estrogen receptors; hence, this mechanism also might have led to an increase in egg production.

However, there have been very few reports on the association of ghrelin and leptin hormones in association with their activity on liver through their receptors during heat stress affecting production. Literature is not available with respect to the modulation of expression of mentioned hormone receptors in chickens subjected to heat stress. A supplement like FYC may maintain hormone levels and receptors' expression without causing a decrease in feed intake and production performance during HS.

Supplementation of FYC decreased the concentration of liver AMP kinase enzyme and was also less in the CR when compared to the CH group on day 20. These findings indicate a deficiency of energy in heat-stressed birds (CH), and to compensate for the moderate decrease in feed intake, the concentration of liver AMP kinase enzyme increased. Similar results have been reported [56]. AMP kinase enzymes integrate nutritional and hormonal signals to promote energy balance by switching on catabolic pathways and switching off ATP-consuming pathways [57]. In response to stresses, ATP depletes, and the intracellular AMP : ATP ratio increases, resulting in the activation of AMPK [58, 59]. Heat production may enhance hyperthermia, which may be detrimental to optimum body functions or production performance. Supplementation of FYC maintained metabolism reflecting less requirement of the enzyme.

Heat stress increases malondialdehyde level and decreases ROS-scavenging enzymes' activities and the levels of natural antioxidants in poultry [60, 61]. YC supplementation decreased plasma MDA level. Heat stress causes hypoxic conditions, leading to less oxygen availability, and stress also leads to more ROS production, as evident by increased plasma MDA levels in the CH group compared to the other two groups. The increase of MDA concentration indicates damage by ROS [62]. Chronic HS showed a 1.2- to 1.5-fold increment in MDA [63, 64]. In the present experiment, also MDA levels increased 1.2–1.4-fold/higher increment in the heat-stressed group compared to CR or SH groups, which indicates oxidative and inflammatory conditions. Hence, supplementation of yeast culture also proved to be beneficial as an antioxidant [65]. It has been proposed that the metabolites in yeast fermentation products may help balance the immune and stress responses and reduce free radical generation [30, 66].

The higher ambient temperature did not significantly affect the CH group's plasma cholesterol level compared to the levels of the CR group on day 10 of the experiment but by day 20, supplementation of FYC decreased plasma cholesterol when compared to CH. Cholesterol is a heat stress marker [67] and dropped upon supplementation of FYC. Heat stress contributes to tight junction dysfunction and gross morphological changes that ultimately reduce intestinal barrier function [26, 68]. A higher concentration of circulatory MDA in the CH group might have led to greater severity of necrosis of the jejunum when compared to the other two groups. In turn, this might have disturbed the absorption of nutrients across the digestive tract and the digestion, ultimately affecting production performance as reported by Zhao et al [69]. Mannan oligosaccharide (MOS), present on the outer layer of autolyzed yeast cell walls [70], prevents colonization of the pathogenic bacteria in the gut and improves the functional structure of intestines [71, 72]. It has also been reported that the use of XPC (YC product) can reduce physiological stress indicators in turkeys placed under short-term heat stress [73] and in broilers under long-term heat stress [74]. Thus supplementation of FYC proved to be beneficial in modulating physiological parameters.

Reduced feed intake during heat stress is an attempt to decrease metabolic heat production [75]. Heat stress induces an altered endocrine status and increased maintenance requirements [76], which results in a decrease in feed intake and energy available for production. The results revealed that the SH group birds consumed more feed from 21 to 25 weeks of age during and after exposure of the experimental tenure. An increase in feed intake might have led to better nutrient absorption, the balance of energy resulting in higher body weight of SH group birds, during and after exposure. Boswell and Dunn [77] affirm that the decline in food consumption occurs due to the heat's inhibitory action at the appetite center due to the high respiratory rate and reduced motility of the gastrointestinal tract, and this may be the reason for less FI in the CH group of birds.

During and after exposure to HS, the body weights of supplemented group birds were more when compared to the CR and CH groups of birds. This indicates that HS for 4 h for three weeks did not affect the body weight of the CH group. A decrease in feed intake and increase in the level of heat stress markers affected egg production only. A significant decrease in feed intake did not translate into decrease in BW but supplementation of FYC increased the FI and BW, compared to the other two groups. Since shortly after the postexposure period, when the laying period began and birds started laying eggs, the stress of the exposure and laying persisted in the CH group, leading to decreased egg production, and FYC supplementation overcame both stresses. Similar reports are available with respect to chickens reared in either natural or controlled environments [6, 13, 78–80]. Similarly, Christopher et al. [81] reported a 32.7% decrease in laying rate (82.9 versus 55.8%) in heat-stressed laying hens compared with hens reared under optimal thermal conditions. Similar results have also been reported in ducks [82]. A study conducted by Attia et al. [10] revealed that plasma hormones and the laying rate decreased in hens reared under heat stress when compared to the supplemented groups. FYC supplemented group delivered better results when compared to the CR group in terms of egg production in the shed even in the postexposure period. In our previous study [39], exposure to higher cyclic ambient temperature during summer decreased egg production in the nonsupplemented group compared to the FYC supplemented group. In the present study, the trend for the egg production performance was CH < CR < SH.

## 5. Conclusion

In conclusion, an indigenous bird-like Nicobari changed its endocrine, metabolic, and gene expression parameters when subjected to higher ambient temperature (39°C for 4 h). These heat stress effects were observed in terms of loss of production performance in the postexposure period also. The current study indicates that hormone levels, receptors expression, and metabolite levels can have an impact on the postexposure period. However, a supplement like FYC (700 mg/kg) provides a tool to negate adverse effects on production performance's by modulating physiological parameters.

## Figures and Tables

**Table 1:** Composition of feed (g/kg) for layers^a^.

Components of diet	Layer grower	Layer breeder
Maize	560.5	613.9
Soybean meal	240.9	247.4
DORB^b^	153.0	4.9
Stone grit	18.6	109.0
DCP^c^	16.6	15.0
Salt	3.5	3.5
Sodium bicarbonate	1.0	1.0
DL-methionine	1.1	1.0
L-lysine	0	0
Trace minerals	1.5	1.0
Vitamin premix	0.25	2.5
Choline chloride	1.0	1.0
Toxin binder	1.0	0
Tylosin	0.5	0
Coccidiostat	0.5	0

^a^Panda et al. [38]. ^b^DORB: deoiled rice bran. ^c^DCP: dicalcium phosphate.

**Table 2:** Sequence of the primers.

Genes	Sequence of forward (F) and reverse (R) primers	Size of the PCR product (bp)	Annealing temperature (oC)
Leptin R	F 5′-CGGACTACCTCATGAAGATCCTGAC-3′	162	55
	R 5′-GCCAATGGTGATGACCTGACCATC-3′		

GSHR	F 5′-CTGCAAGCTCTTCCAGTTCATCAGC-3′	158	55
	R 5′-CCAGAGGATGAGGATGACCAGCTTG-3′		

GH R	F 5′-TCAGAAAGGATGGATTACTCTGGAGTATG -3′	161	55
	R 5′-CGGAGGTACGTTGTCTTGATCGGAC-3′		

Actin *β*	F 5′-CGGACTACCTCATGAAGATCCTGAC-3′	197	55
	R 5′-GCCAATGGTGATGACCTGACCATC-3′		

**Table 3:** Mean levels of hormones, metabolites, and liver enzymes of Nicobari chickens subjected to different treatments.

Days 1	CR^2^	CH^2^	SH^2^
	Plasma leptin (ng/mL)		
10	3.34^a^ ± 0.14	1.04^b^ ± 0.12	3.83^c^ ± 0.11
20	3.66^a^ ± 0.12	1.81^b^ ± 0.09	4.09^c^ ± 0.10

	Plasma ghrelin (pg/mL)		
10	61.39^a^ ± 0.92	30.58^b^ ± 0.81	62.27^a^ ± 1.34
20	75.74^a^ ± 0.75	55.91^b^ ± 0.87	63.86^c^ ± 1.5

	Plasma GH (ng/mL)		
10	3.09^a^ ± 0.12	4.07^b^ ± 0.15	4.12^b^ ± 0.11
20	3.71^a^ ± 0.12	2.83^b^ ± 0.11	3.70^a^ ± 0.14

	Plasma MDA (*µ*M/mL)		
10	11.33^a^ ± 0.6	13.94^b^ ± 0.7	8.25^c^ ± 0.5
20	11.20^a^ ± 0.67	15.20^b^ ± 0.75	10.53^a^ ± 0.80

	Plasma cholesterol (*µ*g/mL)		
10	335.56^a^ ± 6.3	334.03^a^ ± 5.05	420.11^b^ ± 4.99
20	264.78^a^ ± 2.50	309.03^b^ ± 3.99	268.58^a^ ± 2.04

	Liver AMP kinase enzyme (ng/mg)		
10	5.82^a^ ± 0.67	6.48^a^ ± 0.52	5.03^a^ ± 0.60
20	4.07^a^ ± 0.51	5.49^a^ ± 0.45	2.04^b^ ± 0.28

^1^10 and 20 days represent days during exposure from 21 weeks of age at 39^o^C for 4 h under controlled conditions. ^2^CR: control maintained at ambient room temperature, CH: control (0 g FYC/kg), and SH: treatment/supplemented with 700 mg/kg of FYC. CH and SH group birds exposed to 39^o^C for 4 h under controlled conditions from 21 to 23 weeks of age. ^a,b,c^In a row, values with different superscripts are significantly different from each other (*P* < 0.01). Values are represented as mean ± SE. *N* = 6 and 5 for hormone and enzyme studies, respectively.

**Table 4:** Body weight (g) (mean ± SE) of Nicobari chickens subjected to different treatments.

Weeks	CR^1^	CH^1^	SH^1^
21^2^	1168.45^a^ ± 19.5	1164.52^a^ ± 17.55	1179.10^a^ ± 17.81
22	1240.46^a^ ± 15.29	1247.21^a^ ± 11.29	1304.96^b^ ± 14.58
23	1277.03^a^ ± 14.01	1255.01^a^ ± 15.60	1337.23^b^ ± 13.23

Weeks	CR	CH	SH
25^3^	1403.81^a^ ± 7.53	1396.54^a^ ± 13.30	1469.22^b^ ± 17.62
27	1480.11^a^ ± 12.37	1505.30^a^ ± 16.20	1564.31^b^ ± 15.23
29	1545.54^a^ ± 17.62	1540.50^a^ ± 17.93	1634.91^b^ ± 20.02

^1^CR and CH: control (0 g FYC/kg). SH: treatment/supplemented with 700 mg/kg of FYC. CH and SH group birds exposed to 39°C for 4 h under controlled conditions from 21 to 23 weeks of age and also from 25 to 29 weeks after exposure. ^2^From 21 to 23 weeks of age, CH and SH group birds were exposed to 39^o^C for 4 h under controlled conditions. CR: birds exposed to normal ambient room temperature. ^3^From 25 to 29 weeks of age, all the groups were exposed to ambient temperature in the shed after exposure. ^a,b^In a row, values with different superscripts are significantly different from each other (*P* < 0.01). *N* = 10.

**Table 5:** Feed intake (g) of Nicobari chickens subjected to different treatments.

Weeks	CR^1^	CH^1^	SH^1^
21^2^	59.83^a^ ± 1.4	49.27^b^ ± 0.74	58.76^a^ ± 0.75
22	53.80^a^ ± 0.97	48.05^b^ ± 1.01	60.99^c^ ± 1.08
23	52.63^a^ ± 0.49	50.79^a^ ± 1.03	56.01^b^ ± 0.67

Weeks	CR	CH	SH
25^3^	67.8^a^ ± 1.19	63.84^b^ ± 0.94	75.50^c^ ± 1.04
27	94.20^a^ ± 1.02	77.7^b^ ± 0.90	94.55^a^ ± 0.95
29	104.05^a^ ± 1.39	77.03^b^ ± 0.99	96.01^c^ ± 0.85

^1^CR and CH: control (0 g FYC/kg). SH: treatment/supplemented with 700 mg/kg of the FYC. CH and SH group birds exposed to 39^o^C for 4h under controlled conditions from 21 to 23 weeks of age and also from 25 to 29 weeks after exposure. ^2^From 21 to 23 weeks of age, CH and SH group birds were exposed to 39^o^C for 4h under controlled conditions. CR: birds exposed to normal ambient room temperature. ^3^From 25 to 29 weeks of age, all the groups were exposed to ambient temperature in the shed after exposure. ^a,b,c^In a row, values with different superscripts are significantly different from each other (*P* < 0.01). Values are represented as mean ± SE. *N* = 10 in each group.

**Table 6:** Effect of different treatments during exposure^1^ on the production of eggs (percentage) of Nicobari chickens during the postexposure period (laying period)^2^.

Weeks	CR^3^	CH^3^	SH^3^
23	—	—	6.6 ± 0.12
24	7.51 ± 0.11	—	11.42 ± 0.05
25	14.16 ± 0.20	11.10 ± 0.21	17.77 ± 0.06
26	27.85^a^ ± 0.63	18.24^b^ ± 0.58	36.56^c^ ± 0.84
27	45.71^a^ ± 0.89	34.56^b^ ± 1.48	49.52^a^ ± 0.98
28	54.28^a^ ± 1.07	49.20^a^ ± 0.97	61.9^b^ ± 0.96
29	57.58^a^ ± 0.77	55.89^a^ ± 0.71	64.23^b^ ± 0.72
30	59.63^a^ ± 0.85	57.23^a^ ± 1.32	70.32^b^ ± 0.74

^1^From 21 to 23 weeks of age, the CH and SH group birds were exposed to 39°C for 4 h under controlled conditions. CR: birds exposed to normal ambient room temperature. Mean ± SEM values for a week are represented in percentage. ^2^Mean ± SEM values for a week are represented in percentage. ^3^CR and CH groups: control (0 g FYC/kg). SH group: treatment/supplemented with FYC (700 mg/kg) from 23 to 30 weeks after exposure. ^a,b,c^Values with different superscripts are significantly different at least at *P* < 0.05.

## Data Availability

The data used to support this study are available at https://krishi.icar.gov.in/jspui/handle/123456789/26059.

## Additional Points

Exposure of Nicobari chickens to controlled higher ambient temperature decreased plasma level of hormones, increased heat stress markers, increased severity of necrotic condition of the jejunum, and increased expression of tissue hormone receptors. Supplementation of fermented yeast culture reversed the above effects and improved production performance during and after exposure.
